# Management of Tubercular Retinal Vasculitis: A Case Report From an Endemic Region

**DOI:** 10.7759/cureus.78167

**Published:** 2025-01-29

**Authors:** Ridham Nanda, Asma Jabeen, Bhavani Raina, Gaveshna Garg, Bhavya Kapoor

**Affiliations:** 1 Ophthalmology, All India Institute of Medical Sciences, Vijaypur, Jammu, IND; 2 Obstetrics and Gynaecology, Grant Government Medical College and Sir JJ Group of Hospitals, Mumbai, IND; 3 Optometry, All India Institute of Medical Sciences, Vijaypur, Jammu, IND

**Keywords:** anti-tuberculosis therapy, ocular tuberculosis, perivascular infiltrates, posterior sub-tenon steroid, retinal vasculitis

## Abstract

India, being an endemic country for tuberculosis, has a very high prevalence of pulmonary and extrapulmonary tuberculosis. Various manifestations of ocular tuberculosis have been described, including granulomatous anterior uveitis, serpiginous choroiditis, and retinal vasculitis. We hereby describe the case of a 39-year-old male patient who presented to our clinic with complaints of diminished vision in the right eye for five months. His best-corrected visual acuity was 6/12 in the right eye with normal anterior segment examination. He was misdiagnosed as having central serous chorioretinopathy in the right eye at a primary center. The patient had typical fundus findings of perivascular infiltrates, focal vascular tortuosity, vitreous snowball infiltrates, and cystoid macular edema. Fluorescein angiography was done to evaluate the retinal findings and capillary non-perfusion areas. The patient was referred to a physician for the initiation of anti-tubercular treatment. Posterior sub-Tenon injection of triamcinolone acetonide was given for cystoid macular edema, and laser photocoagulation of capillary non-perfusion areas of the retina was done. The patient responded well to treatment with resolution of macular edema and healing of perivascular infiltrates. His visual acuity improved to 6/6 at the three-month follow-up. The patient was followed up for a period of two years with no reported complications or recurrences. This case highlights the diagnostic features of tubercular retinal vasculitis and its management.

## Introduction

Ocular tuberculosis (TB) is a rare extrapulmonary manifestation of *Mycobacterium tuberculosis* infection, affecting various ocular structures with variable clinical manifestations [1]. The incidence has been reported to be around 1.4%-18%, depending upon the region’s endemicity [2]. It remains a diagnostic challenge, as a significant proportion of patients with extrapulmonary TB do not exhibit pulmonary involvement [3]. Ocular TB can manifest as anterior uveitis, intermediate uveitis, posterior uveitis, panuveitis, or retinal vasculitis, with posterior uveitis being the most common form [1,2]. Retinal vasculitis is typically associated with vitritis, periphlebitis, retinal hemorrhages, peripheral capillary non-perfusion, and potential neovascularization [4]. Subvacular lesions (chorioretinitis lesions underlying retinal vessels), focal vascular tortuosity, and occlusive vasculitis are characteristically described as predictors of tubercular retinal vasculitis (TRV) in endemic countries [5]. Common diagnostic approaches include clinical examination in conjunction with positive tuberculin skin tests (TSTs) or interferon-gamma release assays (IGRAs), such as QuantiFERON-TB Gold, which suggest latent TB infection. Radiological imaging may also be employed, although signs of active pulmonary disease are frequently absent. In some instances, polymerase chain reaction (PCR) analysis of intraocular fluids or tissue biopsies can confirm the diagnosis by detecting mycobacterial DNA; however, these procedures are invasive and not routinely performed [6]. The treatment of ocular TB involves a multifaceted approach. Standard anti-tubercular therapy (ATT) is the cornerstone for managing the underlying infection [7]. Oral corticosteroids are often used as an adjunct to reduce intraocular inflammation. Additional interventions, such as laser photocoagulation, are applied to ischemic retinal areas to prevent neovascularization. Additionally, pars plana vitrectomy may be required for non-resolving vitreous hemorrhage or tractional retinal detachment [8].

## Case presentation

A 39-year-old male patient presented with complaints of progressive, painless, diminished vision in the right eye for the past five months. The patient had no prior history of any systemic illnesses. However, there was a history of exposure to a pulmonary TB patient for one year. On presentation, his best-corrected visual acuity (VA) was 6/12 in the right eye and 6/6 in the left eye, and slit lamp examination revealed a normal anterior segment in both eyes.

**Figure 1:**
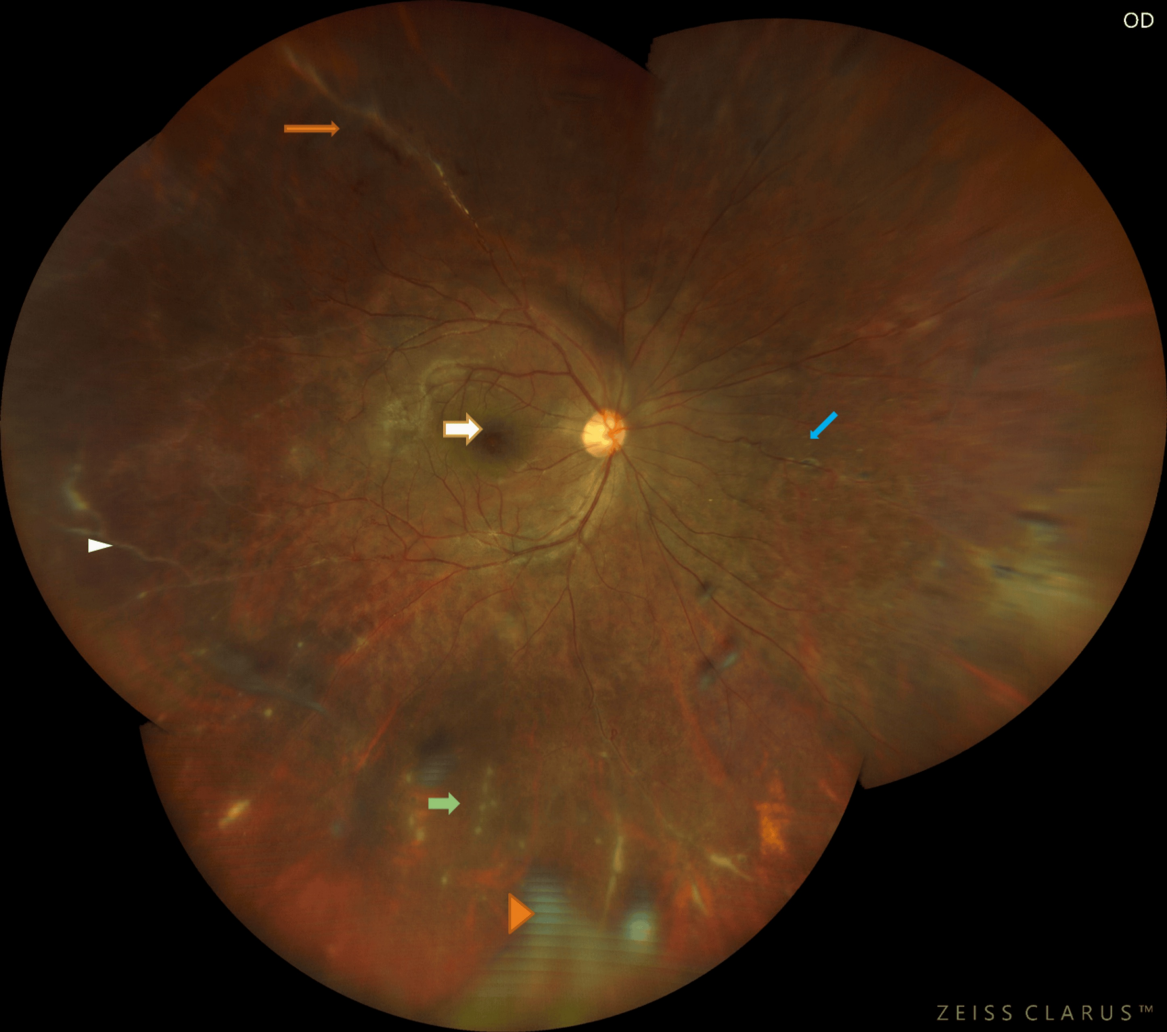
Fundus of the right eye demonstrating snowballs in vitreous (orange arrowhead), macular edema (white arrow), perivasculitis in superotemporal arcade with hemorrhages (confluent vasculitis) (orange arrow), subvascular active infiltrates (green arrow), sclerosed vessels (white arrowhead), and healed subvascular infiltrates (blue arrow).

Fundus examination of the right eye revealed perivasculitis in the superotemporal arcade veins with associated hemorrhages indicative of confluent occlusive vasculitis. Subvascular infiltrates were observed in the third-order veins of the inferotemporal quadrant, along with snowballs in the inferior vitreous. The optic disk appeared well defined, with a cup-to-disk ratio of 0.3. Additional findings included perivascular pigmented chorioretinal atrophic patches in the nasal retina, macular edema, focal vascular tortuosity, and sclerosed vessels in the temporal peripheral retina (Figure 1).

**Figure 2:**
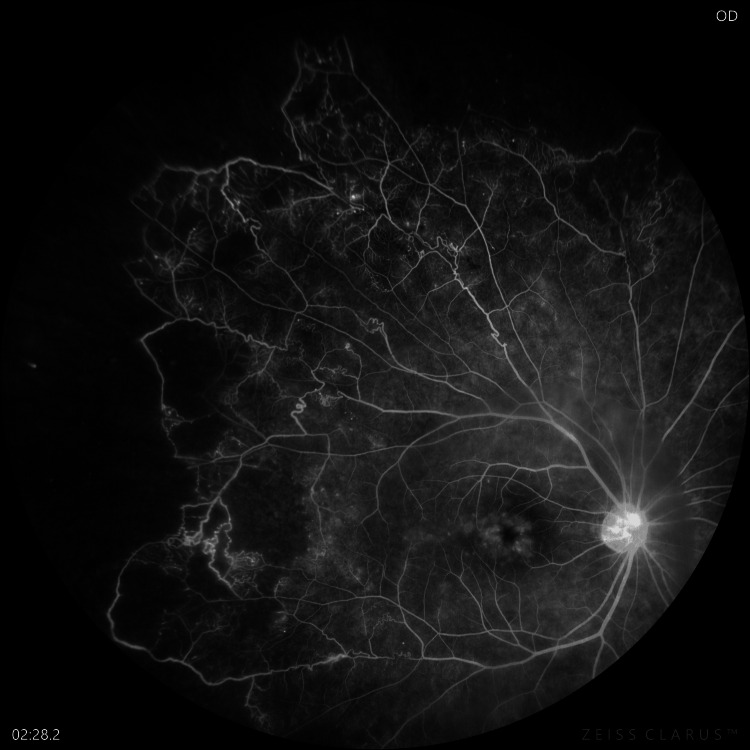
Fluorescein angiography of the right eye showing capillary non-perfusion areas in superior and temporal peripheries, microaneurysms, collaterals, and flower petal pattern at the macula.

Initial blood investigations revealed normal blood counts and hemoglobin levels but an elevated ESR (65 mm/hr). The Mantoux test was strongly positive (23 mm), and IGRA-QuantiFERON-TB Gold demonstrated high positivity (2.402 IU/mL), confirming TB exposure. Serum angiotensin-converting enzyme (ACE), *Treponema pallidum* hemagglutination assay (TPHA), and HIV tests were negative. Imaging, including a chest X-ray and high-resolution computed tomography (HRCT) chest scan, showed no abnormalities. Fundus fluorescein angiography (FFA) highlighted capillary non-perfusion areas, multiple collaterals, and cystoid macular edema (Figure 2).

The patient was initially misdiagnosed as central serous chorioretinopathy elsewhere. We considered a differential diagnosis of syphilis, sarcoidosis, and tubercular posterior uveitis based on the typical fundus findings. We ruled out syphilis as the patient was negative for TPHA. Sarcoidosis was ruled out in view of Mantoux positivity, serum ACE negativity, and absence of pulmonary involvement. The clinical findings, peripheral retinal examination, and systemic investigations led to a final diagnosis of posterior uveitis secondary to TB.

**Figure 3:**
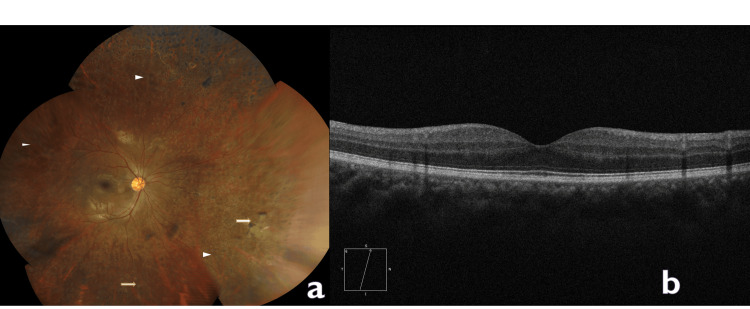
(a) Fundus of the patient at the three-month follow-up showing healed subvascular infiltrates (white arrow) and laser scars (white arrowhead). (b) Normal optical coherence tomography findings with resolution of macular edema.

The patient was managed with ATT and posterior sub-Tenon triamcinolone for macular edema. ATT included two months of intensive treatment with isoniazid, rifampicin, ethambutol, and pyrazinamide and four months of maintenance therapy with isoniazid and rifampicin. Medication was adjusted as per body weight and was administered by an internist. Triamcinolone acetonide, 40 mg (1 mL), was injected superotemporally into the sub-Tenon space beneath the conjunctiva using the technique described by Smith and Nozik [9,10]. Laser photocoagulation of the peripheral retina was done in view of the presence of large capillary non-perfusion areas. The patient was asked to continue the same treatment and follow up monthly. At the one-month follow-up, the patient reported symptomatic improvement, but the best-corrected VA was 6/12 in the right eye with 12 mm Hg intraocular pressure (IOP). The anterior chamber was quiet, and healing of perivascular infiltrates was noted, along with a significant reduction in vitreous exudates. The dose of steroids was gradually tapered, and macular edema showed marked improvement on clinical examination. At three months, the patient’s vision had improved to 6/6 in the right eye with 16 mm Hg IOP. Fundus examination revealed healed pigmented subvascular infiltrates, with evidence of laser photocoagulation marks in the peripheral retina (Figure 3a).

Optical coherence tomography (OCT) findings were within normal limits, confirming the resolution of macular edema (Figure 3b). He was compliant with the prescribed ATT and completed ATT with no complications reported. He was followed up monthly till the completion of ATT and thereafter three-monthly for a period of two years. The patient remained stable (VA-6/6) with IOP in the normal range and no signs of recurrence or active inflammation.

## Discussion

Ocular TB is challenging to diagnose clinically despite the endemicity and availability of various conventional and advanced investigative modalities. The pathogenesis of retinal vasculitis is still debated, as it may result either from direct mycobacterial infection or an immune-mediated hypersensitivity reaction to *M. tuberculosis* antigens [11]. Retinal vasculitis is a manifestation of TB that primarily affects the retinal veins in an occlusive manner [5,12]. Similar to other types of retinal vasculitis, TRV progresses through stages of inflammation, blood vessel blockage, abnormal new blood vessel growth, and traction-related complications. In a retrospective study, Gupta et al. analyzed the ophthalmological findings of 386 patients with uveitis. They concluded that retinal vasculitis, broad-based posterior synechiae, and serpiginous choroiditis were significantly more common in cases of TB uveitis [12].

Ocular TB poses diagnostic challenges with the need to evaluate clinical features and laboratory investigation [7]. The TST and IGRA-QuantiFERON-TB Gold are valuable screening tools for latent TB, particularly in patients with clinical features suggestive of intraocular TB and no indications of an alternative cause [2,4]. In our patient, the presence of vasculitis, hemorrhages, macular edema, and sclerosed vessels, along with strongly positive Mantoux and IGRA tests, confirmed the diagnosis of TB-related retinal vasculitis. The peripheral retinal findings, including capillary non-perfusion and collaterals identified on FFA, highlight the ischemic changes characteristic of this condition. The COTS calculator is an evidence- and experience-based clinical tool to guide ATT initiation in patients with ocular TB [13]. Using the COTS calculator, the patient in our study achieved a median score of five, indicating a very high probability (81%-100%) for initiating ATT, according to expert consensus. This emphasizes the diagnostic complexity and highlights the importance of integrating clinical judgment, systemic findings, and ancillary test results when managing ocular TB.

Studies have documented the potential for retinal vasculitis to progress to neovascularization and vitreous hemorrhage. In a series of 13 PCR-positive cases of TB-related retinal vasculitis, neovascularization was observed in 60% of eyes after vasculitis resolution, and more than half of patients in a more extensive presumed TRV series developed vitreous hemorrhage [14]. If untreated, these complications may lead to tractional retinal detachment, iris neovascularization, or neovascular glaucoma [15].

Our patient was misdiagnosed elsewhere as central serous chorioretinopathy in a peripheral center. Probably, the treating clinician did not perform a complete dilated examination. This case highlights the need for the evaluation of retinal peripheries in patients with macular edema to identify ischemic changes and vascular abnormalities that may be missed during routine assessments. Additionally, our patient responded well to posterior sub-Tenon triamcinolone injection despite having severe cystoid macular edema. It is important to rule out any other infective etiologies before giving periocular steroids. It is pertinent to mention that the IOP of such patients should be monitored regularly, and our patient did not report any increase in IOP.

This case contributes to understanding TRV by highlighting the practical challenges of its diagnosis and management, particularly in endemic regions where the disease burden is high and diagnostic resources may be limited. The presence of snowball infiltrates in vitreous, subvascular infiltrates, occlusive vasculitis, focal vascular tortuosity, and a history of contact with TB patients and positive systemic investigations for TB (Mantoux and IGRA) made the diagnosis of TRV likely. This case emphasizes the importance of integrating clinical judgment, ancillary testing, and tools like the COTS calculator to guide therapy. Globally, it underscores the need for standardized approaches to differentiate TRV from other causes of retinal vasculitis and manage complications effectively. By demonstrating the benefits of early recognition, appropriate treatment, and individualized care, this case provides valuable insights into optimizing outcomes for TRV patients in diverse healthcare settings.

## Conclusions

This case highlights the importance of a detailed fundus examination, including peripheral retina evaluation, in cases of macular edema to avoid misdiagnosis. Retinal vasculitis warrants comprehensive investigations to rule out systemic diseases such as syphilis, sarcoidosis, and Behçet’s disease. The treatment approach for TRV involves a combination of ATT, corticosteroids, and interventions for complications such as capillary non-perfusion and neovascularization.
